# Breast Carcinoma Cells in Primary Tumors and Effusions Have Different Gene Array Profiles

**DOI:** 10.1155/2010/969084

**Published:** 2009-08-11

**Authors:** Sophya Konstantinovsky, Yoav Smith, Sofia Zilber, Helene Tuft Stavnes, Anne-Marie Becker, Jahn M. Nesland, Reuven Reich, Ben Davidson

**Affiliations:** ^1^Department of Pharmacology and Experimental Therapeutics, School of Pharmacy, Faculty of Medicine, The Hebrew University of Jerusalem, Jerusalem 91120, Israel; ^2^Genomic Data Analysis Unit, The Hebrew University of Jerusalem, Jerusalem 91120, Israel; ^3^Department of Pathology, Rabin Medical Center, Petach Tikva 49100, Israel; ^4^Division of Pathology, The Norwegian Radium Hospital, Oslo University Hospital, 0310 Oslo, Norway; ^5^Faculty Division Norwegian Radium Hospital, The Medical Faculty, University of Oslo, 0316 Oslo, Norway; ^6^David R. Bloom Center for Pharmacy, The Hebrew University of Jerusalem, Israel

## Abstract

The detection of breast carcinoma cells in effusions is associated with rapidly fatal outcome, but these cells are poorly characterized at the molecular level. This study compared the gene array signatures of breast carcinoma cells in primary carcinomas and effusions. The genetic signature of 10 primary tumors and 10 effusions was analyzed using the Array-Ready Oligo set for the Human Genome platform. Results for selected genes were validated using PCR, Western blotting, and immunohistochemistry. Array analysis identified 255 significantly downregulated and 96 upregulated genes in the effusion samples. The majority of differentially expressed genes were part of pathways involved in focal adhesion, extracellular matrix-cell interaction, and the regulation of the actin cytoskeleton. Genes that were upregulated in effusions included *KRT8, BCAR1, CLDN4, VIL2*, while *DCN, CLDN19, ITGA7*, and *ITGA5* were downregulated at this anatomic site. PCR, Western blotting, and immunohistochemistry confirmed the array findings for *BCAR1, CLDN4, VIL2*, and *DCN*. Our data show that breast carcinoma cells in primary carcinomas and effusions have different gene expression signatures, and differentially express a large number of molecules related to adhesion, motility, and metastasis. These differences may have a critical role in designing therapy and in prognostication for patients with metastatic disease localized to the serosal cavities.

## 1. Introduction

Breast cancer is the most common malignancy in women, constituting more than 25% of cancers in this group. The incidence of breast cancer around the world greatly varies, with highest rates in the North America, Western, and Northern Europe and Australia/New Zealand (82.5–99.4 per 100,000 women). Mortality in these areas of high incidence is 19.2–22.6 per 100,000 women [1]. Breast cancer metastasizes most often to axillary lymph nodes, but may involve any organ. Metastasis to serosal surfaces involves primarily the pleural cavity [2, 3], and infrequently, the pericardial and peritoneal cavities [4]. Pleural effusions may occur at any point of time in the clinical course and may be the sole manifestation of metastatic disease [5]. This condition is associated with a poor median survival of less than 1 year [5, 6]. 

 In recent years, we have reported on the differential expression of metastasis-related molecules, including proteases, angiogenic molecules, signaling molecules, inhibitors of apoptosis, and transcription factors, in breast carcinoma effusions compared to patient-matched primary carcinomas [7–10]. However, these studies focused on a limited number of genes. The global view of cellular transcriptional activity using gene array technology is required to identify clusters of genetic markers that explain the complex biological processes involved in carcinoma progression to effusions. At present, only one study in which breast carcinoma effusions were compared with primary carcinomas is available. Dupont et al. analyzed the gene expression signature of 19 effusions and compared them to 4 primary carcinomas, 8 cells lines and 4 specimens consisting of benign breast tissue. Effusions could be differentiated into two categories, one resembling cells lines and expressing CD24, CD44 and cytokeratins 8, 18 and 19, the other expressing metastasis-associated genes, such as S100A4, uPA receptor, vimentin and CXCR4 [11]. 

 The present study compared the gene expression signatures of 10 effusions and 10 primary breast carcinomas. Selected differentially expressed genes of pathways related to adhesion, interaction with the extracellular matrix (ECM) and regulation of the actin cytoskeleton were validated on mRNA and protein level, and their clinical relevance was analyzed in a larger series of breast carcinoma effusions. Our data demonstrate that in agreement with our previous observations, breast carcinoma cells in effusions are markedly different from their counterparts in primary carcinomas. This bears relevance to validation of novel therapeutic targets and stratification of patients with respect to treatment response and survival.

## 2. Materials and Methods

### 2.1. Patients and Material

#### 2.1.1. Effusions

Ten effusions (7 pleural, 2 peritoneal, 1 pericardial) from patients with primary breast carcinoma (9 of infiltrating ductal type, 1 lobular) were submitted for routine diagnostic purposes to the Department of Pathology at the Norwegian Radium Hospital during the period 1999–2005. Submitted specimens were processed immediately upon arrival, and pellets were used for preparation of paraffin-embedded cell blocks and for freezing in equal volumes of RPMI supplemented with 20% fetal calf serum and 20% DMSO. All specimens underwent morphological evaluation and were further characterized using immunohistochemistry, as previously detailed [12]. All effusions had >50% carcinoma cells of the total cellular content, ranging between 80–100% of cells in 8/10 specimens. Study approval was given by the Regional Committee for Medical Research Ethics in Norway.

#### 2.1.2. Primary Tumors

Ten primary breast carcinomas of infiltrating duct type were retrieved from snap-frozen archival material stored at −70°C at the Department of Pathology, Norwegian Radium Hospital. Morphological evaluation of frozen sections from the studied specimens was performed in all cases in order to ensure the presence of a predominant carcinoma cell population and absence of necrosis.

### 2.2. Gene Array

Eleven samples were prepared from each group (one effusion and one primary carcinoma with two samples each). Analysis of differentially expressed genes was performed by two experimental designs. (1) Eleven pair-wise competitive hybridizations of cDNA target sample from randomly chosen patients from the effusion group and sample from randomly chosen patients from primary solid tumor group. (2) Six pair-wise hybridizations of cDNA samples from randomly chosen patients from the effusion group and pooled reference sample from four primary solid tumors. 

#### 2.2.1. RNA Extraction

Biopsies from primary tumors and effusions were lysed using SV- total isolation kit (Promega, Madison, Wis, USA) and total RNA was extracted according to the manufacturer's guidelines. The quantity and quality of the total RNA preparations were assessed using a NanoDrop ND-1000 (NanoDrop, Wilmington, De, USA) combined with agarose gel electrophoresis and only samples with ribosomal 28S/18S ratio near 2 were selected for array analysis.

#### 2.2.2. Reverse-Transcription Reaction

Twenty micrograms total RNA was mixed with 2 *μ*g oligo dT (Amersham Biosciences, Piscataway, NJ, USA) and RNAse-free water to a total volume of 16.9 *μ*L, incubated 10 minutes at 75°C and cooled on ice. Six microliters of first strand buffer, 3 *μ*L of 0.1 M DTT, 2 *μ*L of Superscript II RT (Invitrogen, Carlsbad, Calif, USA), 1.2 *μ*L 25X Aminoallyl (aa-aUTP)/d-NTP mix (Sigma, Haverhill, UK) and 1 *μ*L of RNAse inhibitor (Amersham Biosciences) were added to each reaction tube. The mixture was centrifuged briefly and incubated 60 minutes at 42°C. Additional 1 *μ*L of Superscript II RT was added following incubation for 60 minutes at 42°C.

#### 2.2.3. RNA Hydrolysis

Free RNA was disassembled with mixture of 10 *μ*L 1 M NaOH and 10 *μ*L 0.5 M EDTA and underwent neutralization with 25 *μ*L 1 M Hepes.

#### 2.2.4. cDNA Purification

Unincorporated aa-aUTP and free amines were removed using Microcon YM-30 spin column (Millipore, Bedford, Mass, USA) and cDNA yield were measured by Nanodrop spectrophotometer. The sample was dried at RT using speed-vac.

#### 2.2.5. Coupling Cyanine Dye Ester to aa-cDNA

The cDNA target samples were labeled by Cy3 or Cy5 fluorescent dyes (Amersham Biosciences) according to the sample group and resuspended in 0.1M Carbonate buffer (pH 8.6). The samples were incubated in the dark for 1 hour in RT and diluted in 35 *μ*L NaOAc 100 mM (pH 5.2). The free dyes were removed using QIAquik PCR clean up kit (Qiagen, Valencia, CA, USA), washed with 5 mM phosphate buffer (pH 8.0, 80% ethanol) and eluted with 4 mM Phosphate elution buffer (pH 8.5). The hybridization efficiency and the yield of the dye incorporation were calculated using Labeled cDNA Calculator (http://www.pangloss.com/seidel/Protocols/percent_inc.html). Equal amount of Cy3/Cy5 labeled cDNA were mixed and dried in the speed-vac.

#### 2.2.6. Hybridization

The slides, with printed Array-Ready Oligo set for the Human Genome Version 3.0 (Qiagen) containing 34,580 longmer probes, representing 24,650 genes and 37,123 gene transcripts, were blocked with 1%BSA in 0.1% SDS 5XSSC buffer, washed in distilled water and dried at 1000 rpm for 2 minutes. The mixed pair of samples was resuspended in hybridization buffer (25% Formamide, 0.1% SDS in 5XSSC) supplied with 0.02 *μ*g t-RNA and denatured at 95°C. The arrays were hybridized in a water bath, in sealed, watertight hybridization chambers (DieTech, Ford City, Pa, USA) for 16–18 hours at 42°C. After hybridization, the slides were rinsed in a coupling jar containing 2 × SSC 0.1% SDS, followed by washing for 5 minutes in 1 × SSC, then for 5 minutes in 0.2 × SSC, and finally for 10 minutes in 0.05 × SSC. The slides were dried as described above. The final visualization is carried out using Axon 4000B fluorescence scanner. Griding and analysis of images was performed using Gene Pix 6.0 software package (Molecular Devices, Sunnyvale, Calif, USA). Specific genes were characterized according to fluorescence intensity of Cy3/Cy5 Dyes. The fluorescent intensities of Cy5 and Cy3 for each target spot were adjusted so that the mean Cy3/Cy5 ratio of housekeeping genes was equal to one, a design allowing more precise analysis of differentially expressed genes.

#### 2.2.7. Statistics

Statistical analysis of microarrays was preformed by The Genomic Data Analysis Unit of Hadassah Medical School, Hebrew University of Jerusalem. 

 The quality assurance, calibration, data normalization (Lowess) [13] and Volcano plot for GPR files were performed by custom built package written in MATLAB R2007a. An additional statistical analysis and clustering were carried out using the Spotfire (Somerville, MA) and Partek (St. Louis, MO) software packages.

 Gene annotations and specific pathways were fingered out using online free access programs such as: GO annotation (http://www.geneontology.org/), Onto-Express, Pathway-Express (Intelligent Systems and Bioinformatics Laboratory, Computer Science Department, Wayne State University).

### 2.3. Reverse Transcription Polymerase Chain Reaction (RT-PCR)

#### 2.3.1. Reverse-Transcriptase Reaction

Total RNA from specimens analyzed for *BCAR1, VIL2* and *DCN* expression was extracted using Tri-Reagent (Sigma) according to the manufacturer's guidelines. 0.5 *μ*g total RNA was reverse-transcribed using the M-MLV Reverse Transcriptase (Promega) with incubation of 2 hours at 37°C, followed by 5 minutes at 95°C, and diluted to 1 : 5 with RNase-free water.

#### 2.3.2. Semiquantitative RT-PCR

RT-PCR was performed on complementary DNA samples using a DNA thermal cycler (Eppendorf Mastercycler gradient, Eppendorf, Hamburg, Germany) with Reddymix PCR master mix (ABgene, Surrey, UK). Primer sequence was as follows: 

*BCAR1.* sense 5′-GGG-CCA-CAG-GAC-ATC-TAT-GAT-3′, antisense 5′-GAG-GAA-CGT-CGT-AGA-CTG-CG-3′ (amplicon size, 318 base pairs [bp]). *VIL2*. sense 5′-GTT-TTC-CCC-AGT-TGT-AAT-AGT-GCC-3′, antisense 5′-TGC-CTT-TGC-AAA-GCT-TTT-ATT-TCA-3′ (amplicon size, 995 bp).

Conditions were as follows: *BCAR1*: 95°C for 3 minutes, denaturation at 95° for 15 seconds, annealing at 59° for 30 seconds, extension at 72° for 20 seconds, 34 cycles; *VIL2*: 95° for 3 minutes, denaturation at 95° for 15 seconds, annealing at 64° for 30 seconds, extension at 72° for 20 seconds, 33 cycles. The HT-1080 fibrosarcoma cell line served as a positive control in both reactions. 

 Products were separated on 1.5% agarose gels, isolated using the Invisorb Spin DNA extraction kit (Invitek GmbH, Berlin, Germany) and sequenced. Gels were photographed by the Kodak EDAS 290 system (Kodak, Rochester, NY, USA). Densitometer analysis of films was performed using a computerized image analysis (NIH IMAGE 1.63) program. *BCAR1* and *VIL2* mRNA levels were established by calculating the target molecule/28S ratio (all cases scored for band intensity compared to control). Expression intensity of 5% or less of control levels was interpreted as negative. Measurements were made at the linear phase of the reaction.

#### 2.3.3. Quantitative RT-PCR (qRT-PCR)

qRT-PCR was preformed using the Mx3000P QPCR System (Stratagene, Calif, USA). Oligonucleotide primers were designed in the Primer Express program (Applied Biosystems, Foster City, Calif, USA). Primer sequences for *DCN* were 5′-TCC-GCT-GAA-GAG-CTC-AGG-AAT-3′ for the forward primer, and 5′-CCT-TGA-GGA-ATG-CTG-GTG-ATA-TTG-3′ for the reverse primer. The primers for RPLPO normalizer gene were: 5′-CCA-ACT-ACT-TCC-TTA-AGA-TCA-TCC-AAC-TA-3′ for the forward primer and 5′-ACA-TGC-GGA-TCT-GCT-GCA-3′ for the reverse primer. One of the primers in each primer pair was designed in exon-exon boundaries region in order to minimize the DNA contamination noise. The specificity of primer binding was analyzed by BLAST (http://blast.ncbi.nlm.nih.gov/) with Human genomic + transcript (Human G + T) database for highly similar sequences (megablast). The primer optimal concentration and the sensitivity, efficiency, and accuracy of qPCR were calibrated by amplifying serial geometric dilutions of pooled sample consisted from five primary tumor and five effusion cDNA samples. 0.1 *μ*g of cDNA product from the Reverse Transcriptase reaction were amplified using DyNAmo SYBR Green qPCR Kit with ROX passive reference dye (Finnzymes Oy, Espoo, Finland) according to the manufacturer's instructions. Absence of primer-dimers and non-specific products was verified by single product peak in the qPCR dissociation curve. In addition, the PCR product was separated by gel electrophoresis and sequenced (Hebrew University facilities).

### 2.4. Immunohistochemistry

Formalin-fixed paraffin-embedded sections were available from 52 breast carcinoma effusions (47 pleural, 4 peritoneal, 1 pericardial) from 51 female patients (one patient with 2 effusions) aged 33–86 (mean = 59) years with histologically verified breast cancer. In 27 cases, the primary carcinoma was additionally available for analysis. 

 Slides from the primary breast carcinoma specimens were available in our archives for 45 cases. These were diagnosed as infiltrating duct carcinoma (38), lobular carcinoma (5) or mixed duct and lobular carcinoma (2). In the remaining 6 cases, effusion specimens were submitted to our clinic from patients operated at other hospitals. 

 These 79 above-described specimens were manually immunostained for p130cas, phospho-Ezrin (p-Ezrin), and claudin-4. The monoclonal mouse p130cas antibody (clone CAS-14) was purchased from NeoMarkers (LabVision Corporation, Fremont, Calif, USA). A monoclonal mouse p-Ezrin antibody was purchased from BD Pharmingen (San Jose, Calif, USA). The rabbit polyclonal claudin-4 antibody was purchased from Zymed (San Francisco, Calif, USA). All slides underwent pretreatment in a microwave oven for 20 minutes (p-Ezrin and claudin-4 slides in Tris/EDTA buffer, pH = 9-9.1, p130^cas^ slides in citrate buffer, pH = 6). Antibody dilutions were 1 : 200 for all antibodies. Visualization was achieved using the EnVision + peroxidase system (Dako A/S, Glostrup, Denmark). 

 Negative controls consisted of sections that underwent similar staining procedures with isotype-matched mouse antibody, normal goat IgG or non-relevant rabbit immunoglobulins according to the antibody host species. Positive controls consisted of a breast carcinoma biopsy that demonstrated immunoreactivity for the studied antigens in a pilot study. 

 Staining was considered positive only when localized to the cell membrane in a linear pattern for p-Ezrin and claudin-4, and when present in the cytoplasm for the p130cas reaction. Staining extent was scored on a scale of 0–4, as follows: 0 = no staining, 1 = staining of 1–5%, 2 = staining of 6–25%, 3 = staining of 26–75%, 4 = staining of 76–100% of cells. No specimen contained less than 100 tumor cells. Slides were scored by a surgical pathologist experienced in effusion cytology and breast pathology (BD). 

### 2.5. Western Blotting

Frozen specimens were thawed and subsequently lysed in 1% NP-40, 20 mM Tris HCl (pH 7.5), 137 mM NaCl, 0.5 mM EDTA, 10% glycerol, 1 mM phenyl-methylsulfonyl fluoride, 1 *μ*g/mL Aprotinin, 2 *μ*g/mL leupeptin, 1 mM sodium orthovanadate, and 0.1% SDS. 25 *μ*g of a total protein from each sample were separated by electrophoresis through SDS-10% polyacrylamide gels under reducing conditions. After electrophoresis, proteins were transferred to Immobilon transfer membranes (Millipore, Bedford, Mass, USA). Membranes were blocked in 5% Non Fat Dry Milk (NFDM) in 0.1% Tween TBS (TBST) and incubated overnight at 4°C in 5% BSA TBST containing anti-p-Ezrin (Thr^567^) rabbit mAb (Cell Signaling Technology Inc., Danvers, Mass, USA). 

 After incubation, membranes were washed and incubated for 1 hour with peroxidase-conjugated AffiniPure goat anti-rabbit IgG (Jackson ImmunoResearch, West Grove, PA, USA) in TBST containing 5% BSA. Membranes were developed using the enhanced chemiluminescence kit (Pierce, Rockford, Ill, USA), according to manufacturer's specifications. Membranes were then washed, stripped in 0.2 M glycine, 0.1% SDS, and 1% Tween 20 (pH 2.2), blocked in TBST containing 5% NFDM, and incubated overnight at 4°C in 5% BSA in TBST containing a rabbit polyclonal anti-Ezrin Ab (Abcam, Cambridge, UK). Total Ezrin activity was normalized to *β*-actin activity measured using rabbit anti-*β*-actin polyclonal antibody (Cell Signaling Technology Inc.). Protein detection was preformed as described above. Protein lysate from MCF-7 breast carcinoma cells served as control. 

 Levels of phosphrylation of the TORC1 substrate p70S6K were analyzed using goat anti-p-p70S6K^Thr389^ and rabbit anti-p70 S6K antibodies (Santa Cruz biotechnology, inc., Santa Cruz, Calif, USA) as described above. Secondary peroxidase-conjugated donkey anti-goat IgG (Santa Cruz biotechnology, inc.) was used for anti-p-p70S6K^Thr389^ detection.

#### 2.5.1. Quantification of Blotting Results

Gels were scanned by the KODAK EDAS 290 system. Densitometer analysis of films was performed using a computerized image analysis program (NIH IMAGE 1.63).

## 3. Statistical Analysis

IHC and IB results were analyzed using the SPSS-PC package, version 15.0 (Chicago, Ill, USA). Comparative analyses of tumor cell expression results in all effusions versus primary tumors were performed using the Mann-Whitney U test. The same test was applied for analysis of the relationship between protein expression in effusions and clinicopathologic parameters. The Wilcoxon Signed Ranks test was applied for patient-matched analysis in the 27 cases with effusion and primary tumor. Univariate analysis for disease-free survival (DFS) and overall survival (OS) for 44 patients with clinical data were executed using the Kaplan-Meier method and Log-rank test. For this analysis, expression categories were grouped as focal (≤25% of cells) or diffuse (>25% of cells).

## 4. Results

### 4.1. Breast Carcinoma Effusions and Primary Carcinomas Have Different Gene Expression Patterns

We have previously shown that effusions constitute a unique form of breast carcinoma metastasis with mRNA and protein expression patterns that differ from primary tumors and solid metastases [7–10]. In the present study, we compared the global expression profile of breast carcinoma cells in effusions with that of primary carcinomas. 

**Figure 1:**
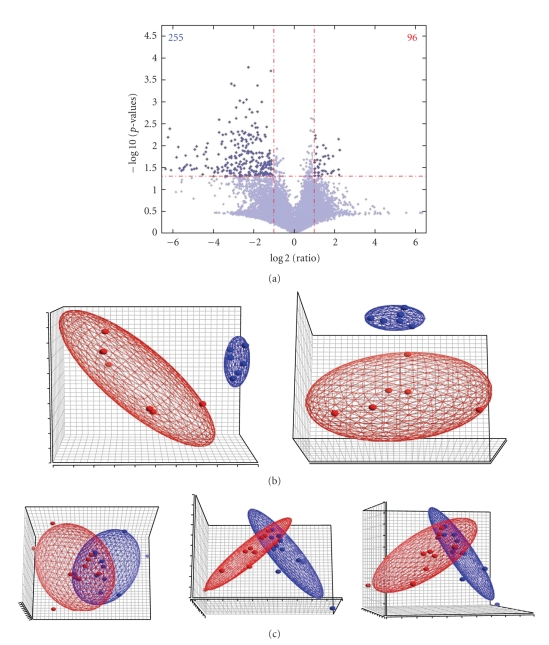
*Breast carcinoma effusion and primary carcinoma expression arrays.* (a)**:** Volcano plot of differentially expressed genes in malignant effusions in comparison to pooled primary tumors (*n* = 6). The x-axis indicates the differential expression profiles, plotting the fold-induction ratios in a log-2 scale. The *y*-axis indicates the statistical significance of the difference in expression (*P*-value, *t*-test) in a -log10 scale. Differentially expressed genes (*P* < .05) appear above the horizontal line. Numbers denote up-regulated (red) or down-regulated (blue) genes in effusions. (b)**:** Gene expression profiling of effusions (blue) and primary breast carcinomas (red) in three-dimensional space by Principal Component Analysis using 351 genes that showed significant up- or downregulation in effusions in comparison to the pooled primary tumor sample (two different view angles). (c): Gene expression profiling of 11 effusions (blue) and 11 primary carcinomas (red) in three-dimensional space by Principal Component Analysis (three different view angles).

Figure 1(a) shows volcano plot of global gene expression in effusions and primary tumors. Differences of 1–5 fold in gene expression with cut-off *P*-value <.05 were defined as significant. We identified 255 significantly down-regulated and 96 significantly up-regulated genes in effusion samples (total = 351). 

#### 4.1.1. PCA Analysis

Principal Component Analysis (PCA) (Partek, St. Louis, Mon, USA) is a technique used to reduce multidimensional data sets to lower dimensions and to highlight their similarities and differences. PCA analysis of six effusion and primary tumor samples was performed using the set of 351 genes that were differentially expressed in effusions and primary carcinomas (Figure 1(b), supplementary Table 1, available at doi:10.1155/2010/969084.). Selected genes are shown in Table 1. The analysis showed that this gene set effectively separates tumors at these two anatomic sites. We additionally performed random PCA analysis of the gene expression pattern in all 11 effusions and 11 primary tumor pairs (Figure 1(c)). The analysis was performed using a set of 342 genes that showed trend of up- or downregulation in those patients. The difference between this gene number and the above-detailed 351 genes results from the fact that two different analyses were performed, the first being a pool versus individual specimen analysis, the second of individual case versus individual case. However, the pathways detected were identical. Three patterns were identified: (1) unique for primary tumors; (2) unique for effusions and (3) samples with overlapping gene expression.

### 4.2. Gene Ontology and Function

In order to understand the biological function of the genes that were up- or down-regulated in effusions, we used the GO annotation (http://www.geneontology.org/) and Pathway-Express (14, 15) programs. 

We found multiple pathways involved in cell maintenance that are altered in effusions in comparison to primary tumors (Table 2). It can be seen that the pathways involved in focal adhesion, ECM-receptor interaction and regulation of the actin cytoskeleton are highly involved in phenotypic transformation of carcinoma cells in primary tumors to those in effusions. Some of the differentially-expressed genes were found to participate in the specific pathways listed above. Other differentially-expressed genes could not be classified as components of a specific pathway, but are of great clinical impact in breast carcinoma, for example, ER*β* with a 3.23-fold downregulation in effusions and MTA3, an estrogen-sensitive gene involved in E-cadherin regulation, with a 2.42- fold downregulation in effusions.

### 4.3. Hierarchical Clustering of Gene Expression Profiles

**Figure 2:**
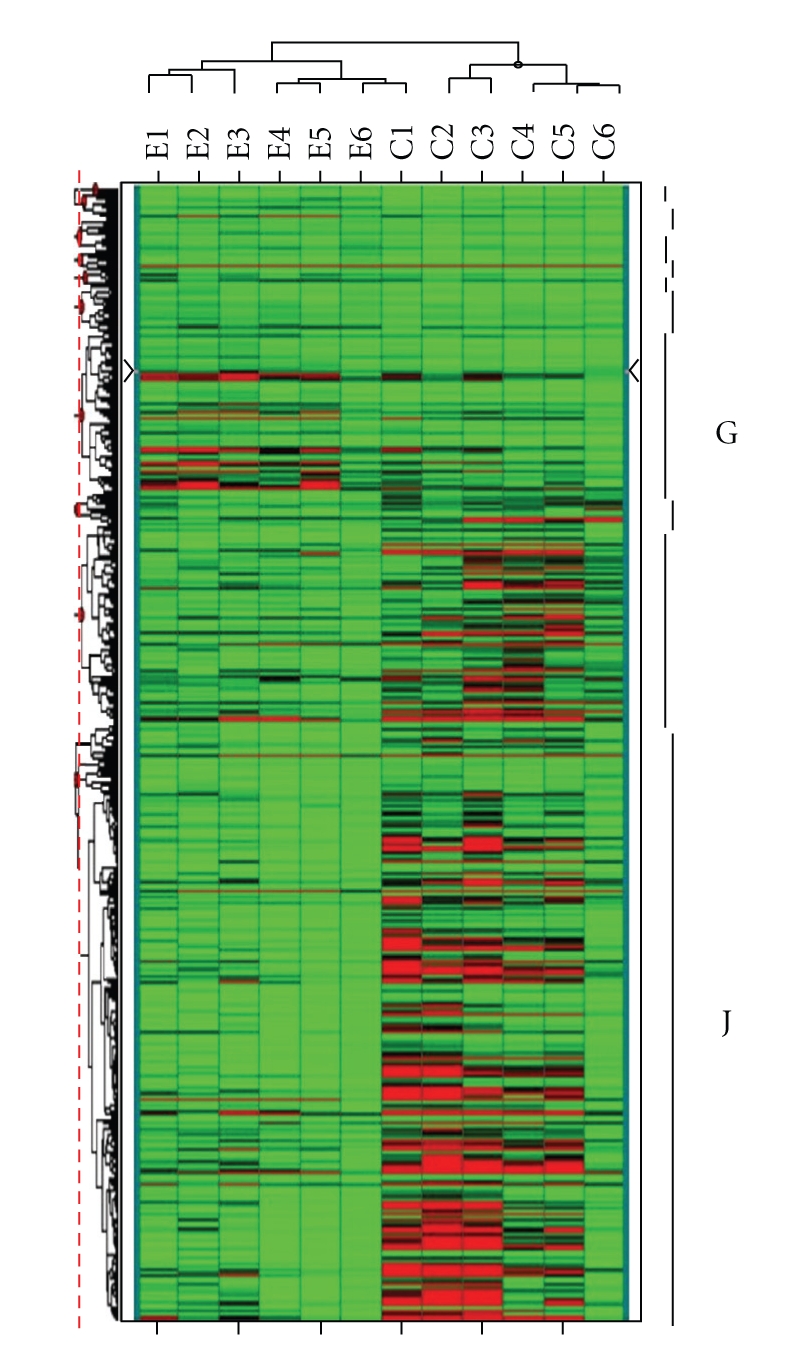
*Dendrogram*. A dendrogram of a supervised analysis of 386 genes (horizontal rows) across 6 effusion versus six primary tumor replicates. Each cell in the matrix represents the expression level of a single transcript in a single sample. The supervised hierarchical clustering analysis clearly distinguished between effusion and control expression pattern. Red or green color indicates high or low transcript levels relative to threshold based on overall gene expression values across each array slide, respectively, while black color indicates equal to median level of expression.

The significantly altered genes in effusions (*t*-test/ ANOVA, *P*-value <.05) were selected for performing UPGMA hierarchical clustering (Figure 2). The unsupervised clustering analysis was performed using the Spotfire software. The differentially expressed genes were classified into 10 major clusters. Of these 10 clusters, cluster G included genes that were strongly up-regulated in effusions, while cluster J genes were strongly down-regulated in comparison to primary tumors. We found some clinically-relevant genes, such as *KRT8*, *CLDN4* and *VIL2* in cluster G, while cluster J included *CLDN19*, the ECM genes *COL1A1*, *COL22A1*, *COL5A2* and the *ITGA7* and *ITGA5* integrin genes.

### 4.4. Validation of Gene Array Results

Since cell motility and cell-ECM interactions may have a major effect on the metastatic potential of carcinoma cells in effusions, we focused on genes participating in regulation of the actin cytoskeleton, focal adhesion, and ECM-receptor interactions in validation of the array results. The genes focused on were the following: *DCN*, which encodes for the small cellular or pericellular matrix proteoglycan decorin [14] and was found to be significantly down-regulated in effusions; *VIL2*, encoding for ezrin, which controls the actin cytoskeleton dynamics; *BCAR*, encoding for the integrin signaling adaptor protein p130cas. In addition, Tuberous Sclerosis 1 (*TSC1*), also known as the tumor suppressor hamartin, the main inhibitor of the mTOR signaling pathway [15, 16], was one of the genes that were found to be down-regulated in effusions in comparison to primary tumors. Thus, we decided to analyze mTOR activity in effusions compared to solid primary tumors. Validation was by semiquantitative and quantitative RT-PCR, Western Blotting and immunohistochemistry, using an enlarged set of effusions and primary carcinomas.

#### 4.4.1. DCN

**Figure 3:**
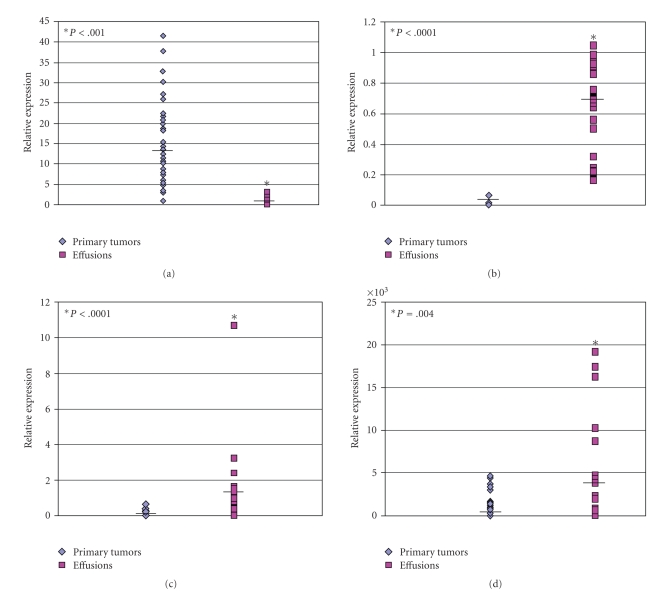
*Validation by PCR and Western blotting*. (a): * DCN* mRNA expression in effusions and solid primary tumors, showing higher expression in the latter specimen type (*P* < .0001). *DCN* levels were measured by qRT-PCR using specific primers for decorin A1, A2 and B isoformes. The pool sample consisting of cDNA from five primary tumors and five effusion samples served as a positive control. *DCN* mRNA expression levels were normalized to RPLPO reference gene expression levels. (b): *BCAR1* mRNA relative expression levels in primary tumors and effusions. Gene expression level was measured by semiquantitative RT-PCR and normalized to relative 28s expression. The MCF-7 breast carcinoma cell line served as positive control. *BCAR1* levels were significantly higher in effusions (*P* < .0001). (c): *VIL2* expression in effusions in comparison to primary carcinomas. *VIL2* mRNA levels were measured by RT-PCR with specific primers and normalized to relative 28s expression. Human HT1080 fibrosarcoma cell line served as a positive control. *VIL2* levels were significantly higher in effusions (*P* < .0001). (d): Total Ezrin protein expression in effusions in comparison to primary carcinomas. Ezrin protein levels were analyzed using specific antibodies and normalized to total beta-actin levels. Ezrin levels were significantly higher in effusions (*P* = .004).

*DCN* expression levels were analyzed in 29 effusions and 35 primary carcinomas using qRT-PCR. *DCN* levels were significantly higher in primary tumors (*P* < .0001, Figure 3(a)), in agreement with the gene array results.

#### 4.4.2. BCAR1/p130cas

**Figure 4:**
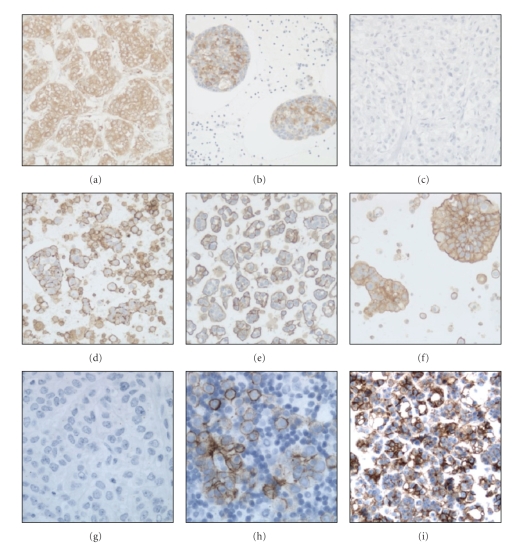
*Validation by immunohistochemistry*. (a)-(b), p130cas: Immunostaining for p130cas in a primary carcinoma (a) and pleural effusion (b) showing cytoplasmic staining in tumor cells. (c)–(f), p-Ezrin: Immunostaining for p-Ezrin in a primary carcinoma (c), pleural effusion from the same patient (d), and 2 additional effusions obtained from the pericardial (e) and pleural (f) cavity. Tumor cells in the primary carcinoma are p-Ezrin-negative, whereas cells in all three effusions are stained at the membrane. (g)–(i), claudin-4: immunostaining for Claudin-4 in a primary carcinoma (g), pleural effusion from the same patient (h), and an additional effusion obtained from the pericardial cavity (i, same specimen as in e). Tumor cells in the primary carcinoma are Claudin-4 -negative, whereas cells in the two effusions are stained at the membrane.

Semiquantitative RT-PCR analysis of 35 primary tumors and of 29 effusions showed significantly higher up-regulation of *BCAR1* in effusions (*P* < .0001, Figure 3(b)). Immunostaining of 52 effusions and 26 of the 27 primary carcinomas (one unsatisfactory reaction) for p130cas showed its presence in tumor cells in 50/52 effusions and 24/26 primary carcinomas (Figures 4(a) and 4(b)). Comparative analysis showed no significant difference in staining extent at these two anatomic sites (*P* > .05). 

**Figure 5:**
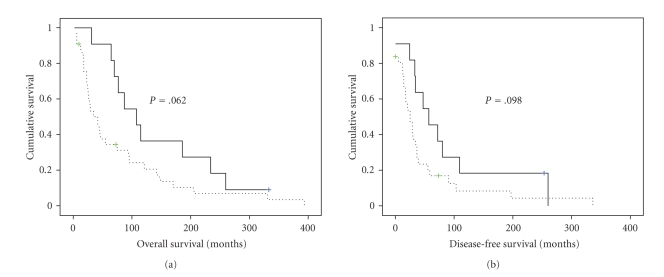
*The prognostic role of p130cas expression in breast carcinoma effusions*. (a): Kaplan-Meier survival curve showing the trend for association between p130cas expression and overall survival (OS) for 44 patients with breast carcinoma effusions. Patients with effusions with higher expression (>25% of tumor cells) (*n* = 33, dashed line) had a mean OS of 78 months versus 142 months for patients whose effusions showed low (≤25%) expression (*n* = 11, solid line; *P* = .062). (b): Kaplan-Meier survival curve showing the trend for association between p130cas expression and disease-free survival (DFS) for 42 patients with breast carcinoma effusions. Patients with effusions with higher expression (>25% of tumor cells) (*n* = 31, dashed line) had a mean DFS of 48 months versus 89 months for patients whose effusions showed low (≤25%) expression (*n* = 11, solid line; *P* = .098). DFS data were unavailable for 2 patients.

 OS for the 44 patients with survival data ranged from 2–393 months (mean = 90 months), while DFS ranged from 0–336 months (mean = 55 months). In survival analysis, higher p130cas expression in effusions was associated with a trend for poor OS (*P* = .062) and DFS (*P* = .098; Figure 5).

#### 4.4.3. VIL2/Ezrin

Semiquantitative RT-PCR analysis of 43 primary tumors and 25 effusions showed significantly higher *VIL2* expression in effusions (*P* = .0021, Figure 3(c)). Protein levels and phosphorylation extent of Ezrin were analyzed by Western blotting using phospho- and pan-specific antibodies. Pan-Ezrin protein level was significantly higher in effusions (*P* = .004, Figure 3(d)), whereas p-ezrin levels did not significantly differ. Although the fraction of phosphorylated protein did not significantly differ, the total amount of the protein was up-regulated in effusions (*P* = .004). Thus, the absolute phosphorylated ezrin levels were higher in effusions compared to primary tumors. 

 Immunostaining of 51 of the 52 effusions (one unsatisfactory reaction) and 27 primary carcinomas showed significantly higher p-Ezrin expression in effusions compared to primary carcinomas (*P* < .001 in analysis of all cases, as well as patient-matched specimens), as evidenced by score = 4 staining in 49/51 effusions and only 2/27 primary carcinomas (Figures 4(c)–4(e)). Ezrin was not analyzed for survival in view of the practically uniform score = 4 staining in effusions.

#### 4.4.4. Claudin-4

Immunostaining of 52 effusions and 23 of the 27 primary carcinomas (4 unsatisfactory reactions) for claudin-4 showed its presence in tumor cells in 51/52 effusions and 20/23 primary carcinomas (Figures 4(f)–4(i)). However, staining extent was higher in effusions, a difference that was significant in analysis of all cases (*P* = .002), and showed a trend in matched specimen analysis (*P* = .062). Claudin-4 protein expression was unrelated to OS or DFS (*P* > .05).

#### 4.4.5. mTOR Pathway Activity

**Figure 6:**
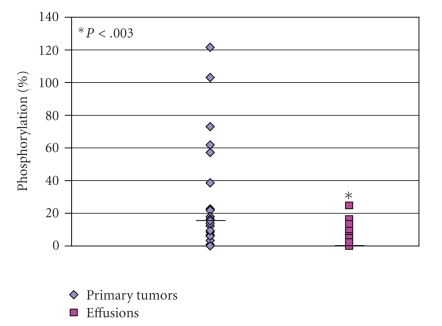
*Reduced p70S6K phosphorylation in breast carcinoma effusions compared to primary tumors*. mTOR activity measured by Western blot analysis of the mTORC1 substrate p70S6K phosphorylation extent in effusions (*n* = 16) and primary tumors (*n* = 22). Phosphorylated p70-S6K protein levels were analyzed using phospho-specific antibodies and normalized to total p70S6K levels. The 3T3-NIH cell line served as a positive control.

*TSC1* showed a 2.3-fold downregulation in effusions compared to primary tumors in the array analysis. Analysis of the phosphorylation level of the rapamycin sensitive mTOR Complex 1 (mTORC1) substrate p70S6K showed that in spite of the *TSC1* downregulation, the levels of p70S6K phosphorylation were lower in effusions compared to primary tumors (*P* = .003, Figure 6).

## 5. Discussion

Breast carcinoma metastasis to the serosal cavities represents an advanced stage in tumor progression and is associated with extensive alterations at the molecular level, involving clinically established targets such as HER-2 and hormone receptors, as well as other cancer-associated molecules [7–10]. Despite the fact that breast carcinoma is one of the most extensively studied cancer forms, little effort has been directed towards understanding the biology of tumor cells in malignant effusions. The major aim of the present study was to characterize a general expression fingerprint that distinguishes effusions from primary tumors. 

 Our data show that 351 genes among 24,650 gene transcripts are significantly altered in effusions in comparison to primary carcinomas. Many of these genes are involved in ECM-receptor interaction, focal adhesion and regulation of the actin cytoskeleton pathways, which define the metastatic potential of carcinoma cells by enhancing their motility and leading to anoikis escape in the absence of ECM molecules. 

 PCA analysis of significantly altered genes distinguished clearly between primary tumors and effusions. The gene expression profile correlated with phenotypic change during the transition of breast carcinoma cells from the solid tumor to suspended cell clusters in pleural effusions. Carcinoma cells in effusions showed down-regulated ECM encoding molecules such as decorin, fibronectin, collagens I, XXII and V, concomitantly with the downregulation of the ECM-binding receptors, integrins *α*5 and *α*7. Thus, it appears as if the cells in effusions lose the requirement for interaction with matrix components, possibly by a compensatory signaling mechanism within the cells. 

 The second goal of the study was to highlight molecules with multiple functional influences on the metastatic potential of cells in effusions. One of these molecules is the cytoskeleton organizer ezrin. This protein provides a functional link between the plasma membrane and the actin cytoskeleton by interacting with the cytoplasmic domains of adhesion membrane proteins and regulation of cytoskeleton polymerization through the Rho pathway activation [17]. Moreover, ezrin promotes growth and survival via AKT/mTOR pathway activation in Ewing's sarcoma cell lines [18]. A recent study provided additional information regarding the role of ezrin in elevation of the metastatic potential of carcinoma cells, by showing that its downregulation of the cell-cell adhesion molecule E-cadherin, and suggesting that ezrin is associated indirectly with the E-cadherin/*β*-catenin complex by regulating Src activation [19]. Screening of a broad spectrum of human cancers, including breast, lung and prostate tumors showed high expression of ezrin in tumors of mesenchymal origin and in primary breast carcinomas. Moreover, ezrin expression was shown to be strongly associated with poor prognosis in breast carcinoma [20]. In agreement with the latter report, higher VIL2 mRNA expression in effusions was associated with poor disease-free survival in our cohort (data not shown). This finding requires further investigation, as it was obtained in analysis of only 17 effusions. 

 The up-regulation of ezrin in effusions was validated using RT-PCR, Western blotting and IHC. We found that the up-regulation of ezrin in effusions is associated with expression of the functionally active T567 phosphorylated ezrin [17, 18] at the plasma membrane. This gene is not only up-regulated at the mRNA and protein levels, but is also more active in effusions compared to solid tumors. Since the increase in ezrin activation may influence multiple metastasis-associated cell functions [17, 18], the therapeutic targeting of this protein may prove beneficial in effusion therapy. 

 Statistical analysis of the array results showed that the *TSC1* gene, encoding a protein involved in mTORC1 inhibition, is down-regulated in effusions in comparison to primary tumors. The mTOR pathway is highly involved in breast carcinoma pathogenesis and adjuvant therapy resistance [21]. Moreover, there is evidence that mTOR activation can lead to anchorage-independent growth of carcinoma cells [22], making it a potentially important factor for cell survival in effusions. Rapamycin analogues, such as temsirolimus (CCI-779) or everolimus (RAD-001) that target mTOR are now in different stages of clinical trials for anti-cancer therapy as a single agent [23] or as additive treatment having synergetic effect with ER- and HER2/neu- targeted therapy [24, 25]. Downregulation of the mTOR inhibitor *TSC1* in effusion may lead to subsequent activation of mTORC1 at this site of metastasis, suggesting that this signaling pathway may be altered along tumor progression in breast carcinoma. 

 Recent studies demonstrate that *TSC1* directly interacts with ezrin and through this interaction regulates focal adhesion complex formation and causes cytoskeletal remodeling [26, 27]. The loss of *TCS1* results in loss of focal adhesions, cell rounding and progressive detachment of cells from the substrate [27]. Since effusions are characterized as clusters of detached carcinoma cells, the parallel dysregulation of *TSC1* and ezrin expression may play a critical role in effusion formation. The relative phosphorylation extent of the mTORC1 substrate p70S6K [28, 29] was measured in order to analyze the effect of TSC1 downregulation on the mTORC1 activity. In spite of the downregulation of the mTORC1 inhibitor *TSC1*, the extent of p70S6K phosphorylation remains low in the effusions in comparison to primary tumors. The reason for low mTORC1 activity in effusions should be investigated. One possible explanation is that other substrates of mTOR may be relevant and p70S6K may be a minor effector of this pathway in effusions. 

 Hormone receptor status and the relevance of adjuvant hormonal therapy at different stages of the disease are central in breast carcinoma research [30]. We have previously shown that ER is down-regulated in effusions in comparison to primary tumors [8]. In the present study we observed *BCAR1* gene up-regulation in effusions. High *BCAR1* expression was reported to be associated with a poor response to first-line tamoxifen therapy in patients with recurrent disease and with an increased rate of relapse [31]. Moreover, overexpression of *BCAR1* causes tamoxifen resistance in tamoxifen-sensitive breast carcinoma cells [32]. The up-regulation of p130cas in effusions can be a result of population enrichment by resistant cells due to tamoxifen treatment. Thus, *BCAR1* expression status in effusions must be taken under consideration while choosing therapeutic regimen in patients with breast carcinoma effusions. On the other hand, p130cas is a downstream effector of integrins [33]. The up-regulation of p130cas can lead to subsequent activation of Rac pathway and actin cytoskeleton rearrangements [34, 35]. This can elevate the metastatic potential of the cells in effusions by enhancing cell migration and leading to anoikis escape, as has been shown in in vitro systems [36, 37]. 

 In contrast to the established importance of ER-*α* as a breast cancer marker, the prognostic and predictive relevance of ER-*β* remains unclear. Several previous reports have shown correlation between low ER-*β* expression and advanced disease stage and shorter survival in various tumors, including gynecological carcinomas [38–40]. In the present study we found downregulation of ER-*β* in effusions. Since breast carcinoma effusions constitute stage IV disease, this observation is concordant with the above-detailed publications. Low ER-*β* levels in effusions may contribute to tamoxifen resistance, as had been shown in ER-positive primary breast carcinomas [41]. Thus, the expression levels of ER-*β* may influence decisions regarding therapeutic regimens for patients with this form of metastatic disease. 

 Claudins are a family of tight junction (TJ)-specific integral membrane proteins, including more than 20 members to date. TJs, located between epithelial or endothelial cells, at the apical region of the adjacent lateral membranes, control the paracellular transport of solutes and maintain cell polarity by blocking the free diffusion of proteins and lipids between the apical and basolateral domains of the plasma membrane [42–44]. TJ filaments also contain occludin, the first TJ-specific integral membrane protein identified, yet it has been shown that claudins are essential and sufficient to form TJ strands [45]. The structure of claudins consists of intracellular amino and carboxy termini, four transmembrane domains, and two extracellular loops mediating interactions between claudins on adjacent cells [42–44]. The second extracellular loop serves as a binding site for *Clostridium perfringens* enterotoxin in claudin-3 and -4 [45]. The carboxy terminus of most claudins contains potential serine and/or thereonine phosphorylation sites and a PDZ-binding motif, to which the TJ cytoplasmic scaffolding proteins ZO-1, -2 and -3 bind [44]. 

 We have recently shown that several claudin family members are upregulated in ovarian carcinoma effusions compared to corresponding primary carcinomas [46]. In the present study, we found upregulation of claudin-4 in breast carcinoma effusions compared to primary carcinomas, suggesting that members of this family are upregulated at this anatomic site in multiple epithelial malignancies. Our observations are in agreement with a recent study in which claudin-4 expression was shown to be associated with high grade and poor prognosis in breast carcinoma [46]. The previously discovered role of claudin-3 and claudin-4 in cell motility and increased MMP-2 activity [47] suggests that this may be yet another metastasis-promoting molecule in breast carcinoma effusions. 

 In conclusion, gene array analysis of breast carcinoma effusions and primary carcinomas showed differences in expression of multiple genes regulating cell motility, invasion and metastasis. The study of effusions and the way they differ from solid tumors will expand our knowledge regarding tumor progression in general, as well as regarding malignancies affecting this anatomic site in particular, and may have an impact on treatment modalities and prognostic models.

## Figures and Tables

**Table 1:** Selected genes identified by fold-change analysis using the MATLAB R2007a program that are differentially expressed in primary tumors versus effusions (complete list available in supplementary Table 1).

Genes more highly expressed in effusions		
Gene	Full name	Log2 change
CLDN4	Claudin-4	4.8544
KRT8	Keratin 8	3.9099
NTN4	Netrin-4	3.5957
VIL2	Villin 2	2.7965
FASN	Fatty acid synthase	2.7583
DLX3	Distal-less homeobox 3	2.3846
ZNF75C	ZNF75C	2.2346
TRAF4	TNF-receptor-associated factor 4	2.0682
NEDD4L	Neural precursor cell expressed, developmentally down-regulated	2.0061
NPC1L1	NPC1 (Niemann-Pick disease, type C1 gene)-like 1	1.9928

Genes more highly expressed in primary tumors		

CLDN19	Claudin-19	20.4327
HPR	Haptoglobin-related protein	11.7846
IGFBP7	Insulin-like growth factor binding protein 7	10.6376
ZFYVE20	Zinc finger FYVE domain containing 20	10.4542
HP	Haptoglobin	9.1384
GFRA1	GDNF family receptor alpha 1	8.6866
APOBEC2	Apolipoprotein B mRNA editing enzyme, catalytic polypeptide-like 2	8.6565
STK39	Serine threonine kinase 39 (STE20/SPS1 homolog, yeast)	8.0239
SLC6A16	Solute carrier family 6, member 16	7.2568

**(a):** 

Pathway name	Number of genes in pathway	Number of differentially expressed genes in pathway	Percentage of differentially expressed genes in pathway	*P*-value
Cell adhesion molecules (CAMs)	132	10	7.576	1.36E-04
Regulation of actin cytoskeleton	208	11	5.288	0.001462
Focal adhesion	195	9	4.615	0.010329
Calcium signaling pathway	175	8	4.571	0.01545
ECM-receptor interaction	87	6	6.897	0.005234
Tight junction	119	4	3.361	0.175458
PPAR signaling pathway	70	3	4.286	0.123277
TGF-beta signaling pathway	84	1	1.19	0.780451
Wnt signaling pathway	149	1	0.671	0.935266
Apoptosis	84	1	1.19	0.792465
mTOR signaling pathway	47	1	2.128	0.584796
Gap junction	92	1	1.087	0.81801
MAPK signaling pathway	256	3	1.172	0.855694
Adherens junction	77	1	1.299	0.763346
Small cell lung cancer	86	1	1.163	0.800108
Cytokine-cytokine receptor interaction	256	1	0.391	0.990004

**(b):** 

Pathway name	Number of genes in pathway	Number of differentially expressed genes in pathway	Percentage of differentially expressed genes in pathway	*P*-value
Cell adhesion molecules (CAMs)	132	9	6.818	4.22E-07
Regulation of actin cytoskeleton	208	4	1.923	0.061989
Focal adhesion	195	3	1.538	0.169683
ECM-receptor interaction	87	2	2.299	0.130698
Tight junction	119	1	0.84	0.580383
TGF-beta signaling pathway	84	5	5.952	3.19E-04
Wnt signaling pathway	149	3	2.013	0.09173
Apoptosis	84	1	1.19	0.46079
mTOR signaling pathway	47	1	2.128	0.291965
MAPK signaling pathway	256	3	1.172	0.288211
Adherens junction	77	1	1.299	0.432252
Small cell lung cancer	86	4	4.651	0.003658
Cytokine-cytokine receptor interaction	256	4	1.563	0.10534
Phosphatidylinositol signaling system	77	2	2.597	0.106703
Pancreatic cancer	73	3	4.11	0.016395
Colorectal cancer	85	3	3.529	0.023709
Cell cycle	114	3	2.632	0.050093
Jak-STAT signaling pathway	153	3	1.961	0.097521
Non-small cell lung cancer	53	2	3.774	0.057405
Endometrial cancer	52	2	3.846	0.055498
Basal cell carcinoma	56	2	3.571	0.059335
Renal cell carcinoma	69	2	2.899	0.09072
Melanoma	71	2	2.817	0.09521
ErbB signaling pathway	87	2	2.299	0.133163
Melanogenesis	102	2	1.961	0.166065
Thyroid cancer	31	1	3.226	0.203583
VEGF signaling pathway	70	1	1.429	0.397797
Prostate cancer	86	1	1.163	0.468679
